# Supplementary material for: Efficacy of Infection Eradication in Antibiotic Cement-Coated Intramedullary Nails for Fracture-Related Infections, Nonunions, and Fusions
Source: Antibiotics (Basel). 2022 May 25;11(6):709. doi: 10.3390/antibiotics11060709 (PMC9219703; doi:10.3390/antibiotics11060709)
Supplement: Supplementary file 1 [file antibiotics-11-00709-s001.zip › antibiotics-1675407-supplementary.pdf]

Efficacy of Infection Eradication in Antibiotic Cement-coated Intramedullary Nails for Fracture-Related Infections, Nonunions, and Fusions

Supplemental Digital Content

Case presentation: 74 year-old male patient who sustained a left ankle open fracture dislocation. Initially it was managed by debridement, open reduction and internal/external fixation, which was complicated by infection. The patient was managed with extensive debridement, bone resection, and ankle fusion using an ACCIN. The final result after 17 months of follow-up was infection eradication and successful ankle fusion.

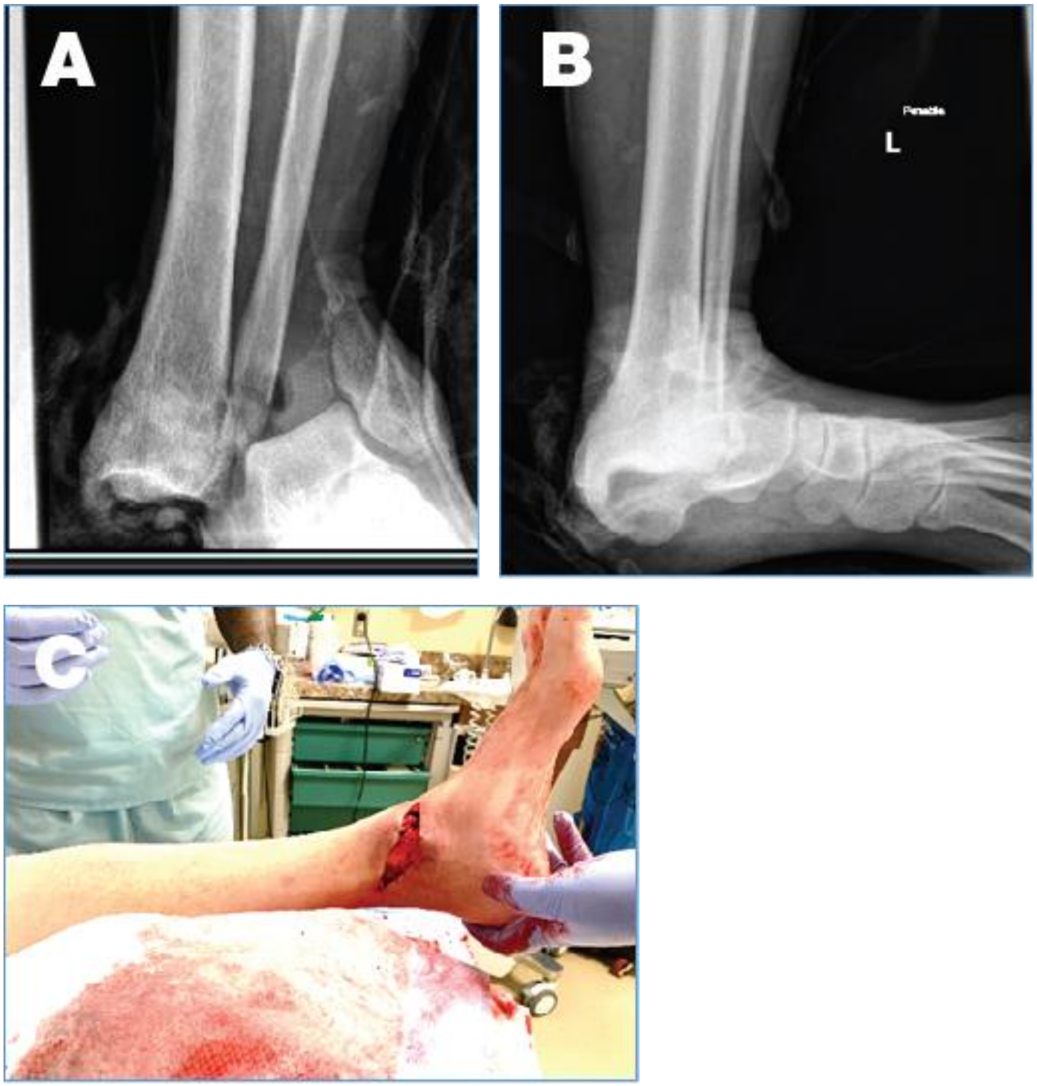

Figure S1. Ankle open fracture dislocation. **A** and **B**: AP and lateral view plain radiographs showing ankle fracture dislocation. **C**: clinical photograph showing a medial wound over the ankle. Copyright 2021, Rubin Institute for Advanced Orthopedics, Sinai Hospital of Baltimore, used with permission.

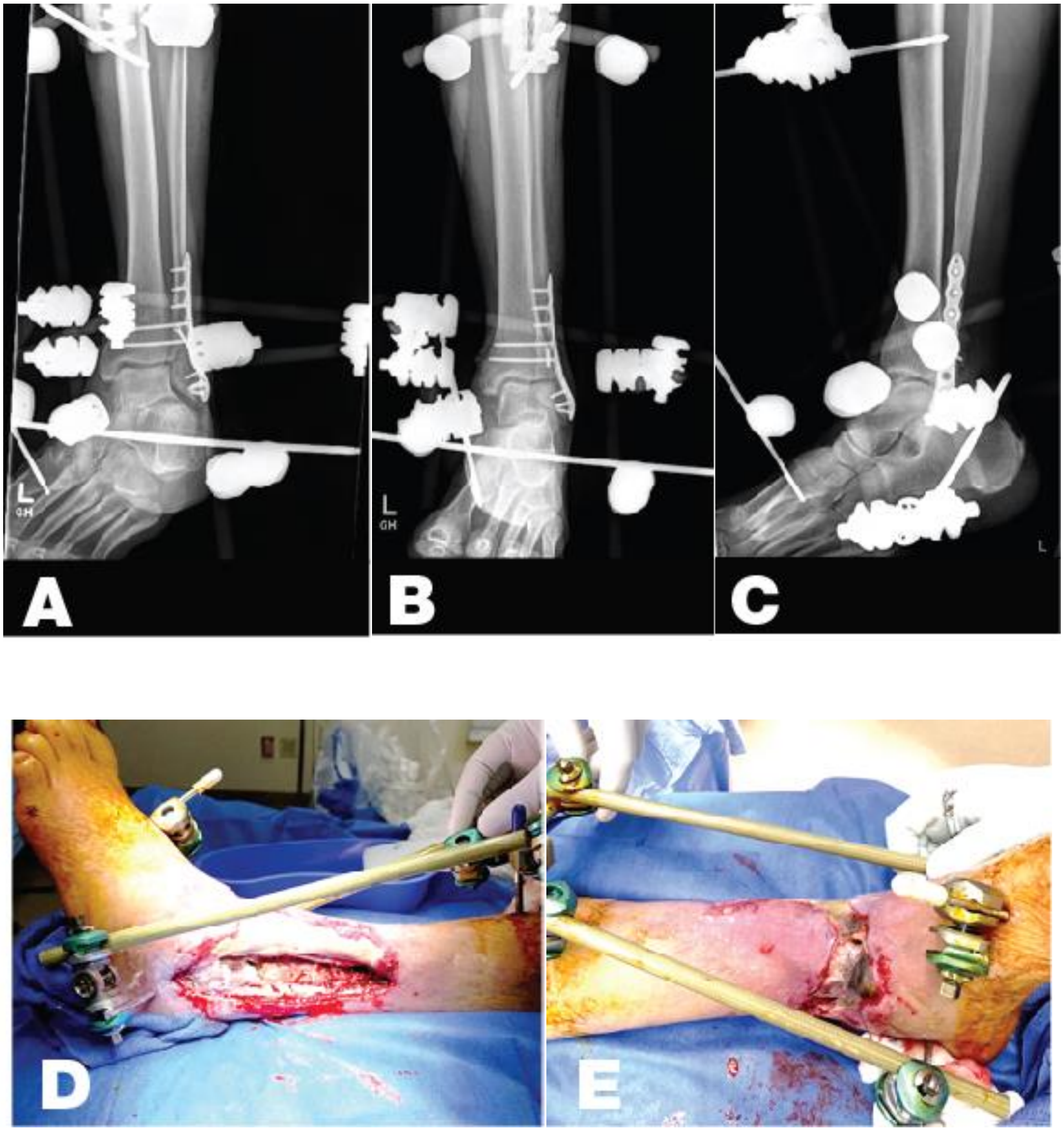

Figure S2. **A**, **B**, and **C**: AP and lateral view plan radiographs taken after the initial open reduction and internal fixation (ORIF) procedure. **D** and **E**, Clinical photos showing infection at the ORIF site after hardware removal, on initial presentation to our institution. Copyright 2021, Rubin Institute for Advanced Orthopedics, Sinai Hospital of Baltimore, used with permission.

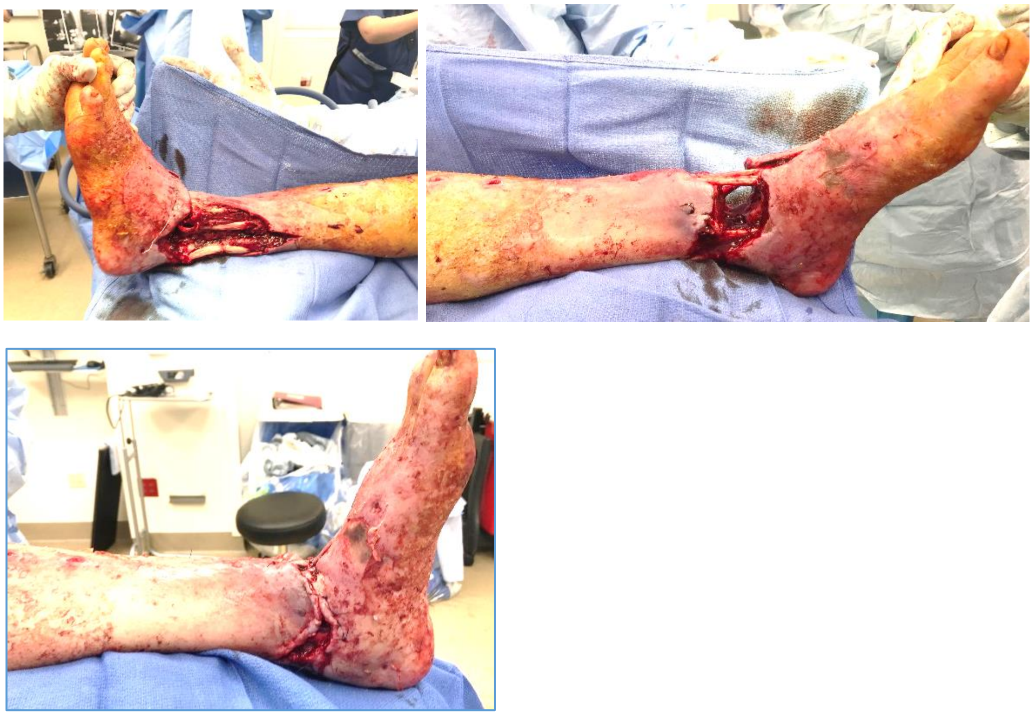

Figure S3. Intraoperative clinical photos after debridement and ankle fusion using an antibiotic cement-coated intramedullary locked nail. Copyright 2021, Rubin Institute for Advanced Orthopedics, Sinai Hospital of Baltimore, used with permission.

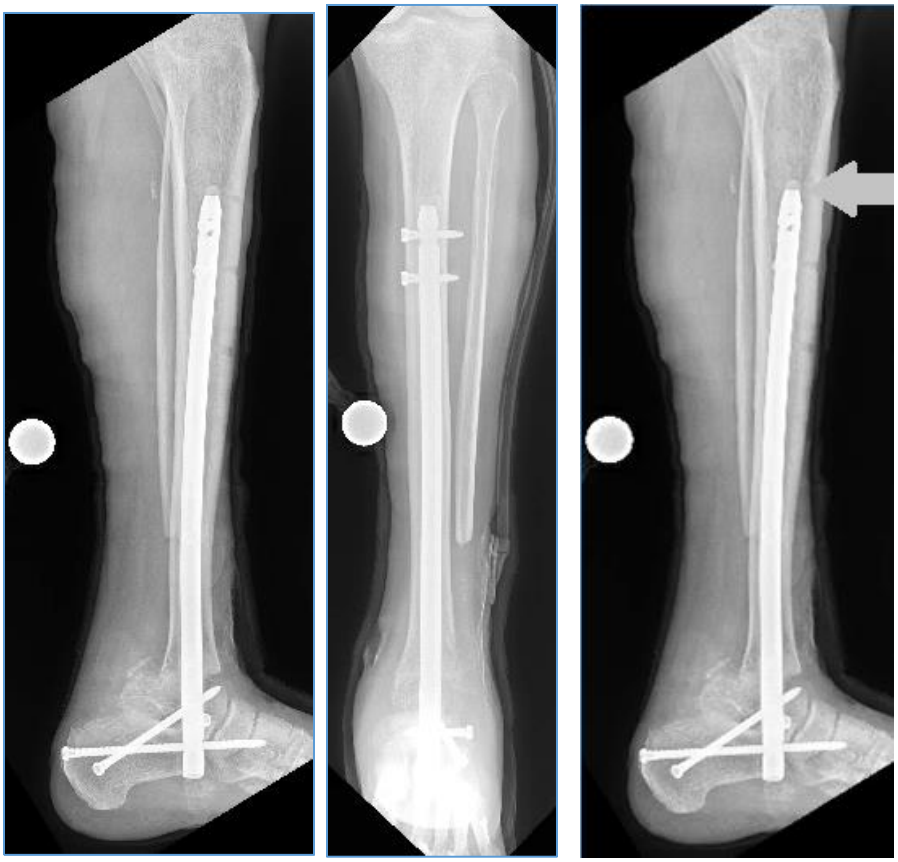

Figure S4. AP and lateral postoperative plain radiographs showing hindfoot dusion using an ACCINs and a missing (excised) distal segment of the fibula. Notice, the cement coating on the the tip of the nail (*gray arrow*). Copyright 2021, Rubin Institute for Advanced Orthopedics, Sinai Hospital of Baltimore, used with permission.

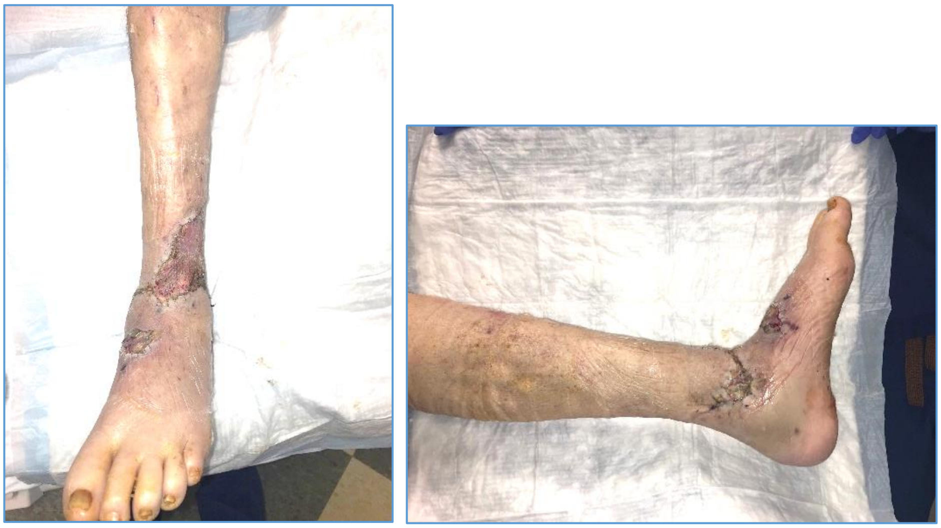

Figure S5. Clinical photos of the leg condition during follow-up. Copyright 2021, Rubin Institute for Advanced Orthopedics, Sinai Hospital of Baltimore, used with permission.

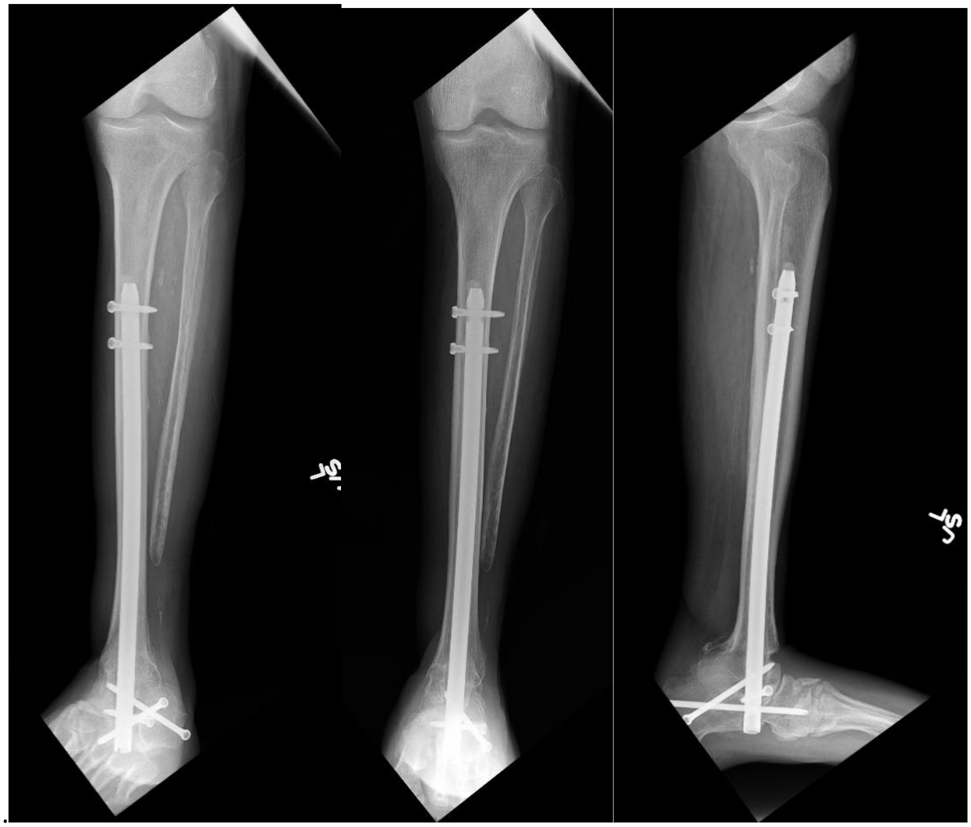

Figure S6. AP and lateral radiographs at the final follow-up showing united ankle fusion at the final follow-up. Copyright 2021, Rubin Institute for Advanced Orthopedics, Sinai Hospital of Baltimore, used with permission.

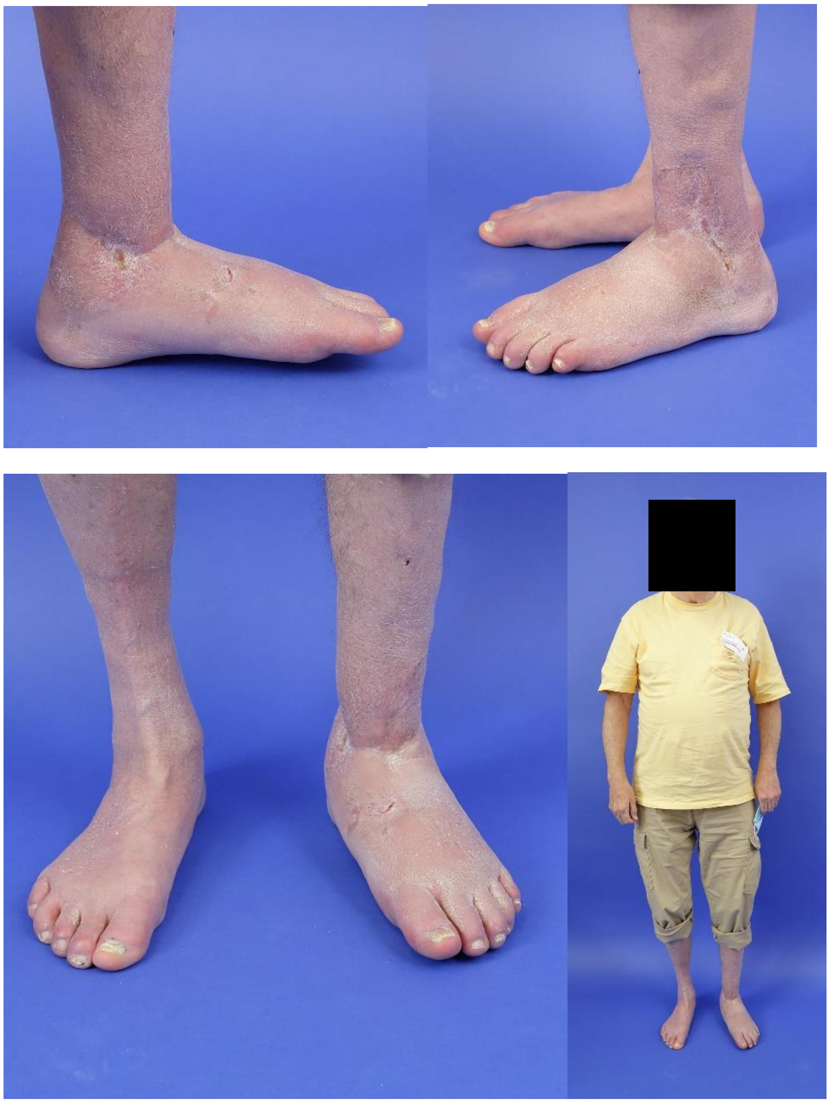

Figure S7. Clinical photographs showing the patient full weigh bearing on both lower limbs. Front and side views of the left foot and ankle showing the skin condition at the last follow-up. Copyright 2021, Rubin Institute for Advanced Orthopedics, Sinai Hospital of Baltimore, used with permission.
